# The Role of Triglycerides in Atherosclerosis: Recent Pathophysiologic Insights and Therapeutic Implications

**DOI:** 10.2174/011573403X272750240109052319

**Published:** 2024-01-26

**Authors:** Yonatan Akivis, Hussam Alkaissi, Samy I. McFarlane, Inna Bukharovich

**Affiliations:** 1 Department of Medicine, SUNY Downstate Health Sciences University, Brooklyn, NY, 11203, USA;; 2 Division of Cardiology, Department of Medicine, NYC Health and & Hospitals, Kings County, Brooklyn, NY, 11203, USA

**Keywords:** Triglycerides, atherosclerosis, cardiovascular disease, lipoprotein metabolism, hypertriglyceridemia, metabolic syndrome, insulin resistance

## Abstract

Triglycerides have long been recognized as a cardiovascular disease risk factor. However, their precise role in atherosclerosis and potential utility as a therapeutic target remains debated topics. This review aims to shed light on these aspects by exploring the complex relationship between triglycerides and atherosclerosis from pathophysiological and pharmacological perspectives.

Triglycerides, primarily carried by chylomicrons and very low-density lipoproteins, play an essential role in energy storage and utilization. Dysregulation of triglyceride homeostasis and triglyceride-rich lipoproteins metabolism often leads to hypertriglyceridemia and subsequently increases atherosclerosis risk. Triglyceride-rich lipoproteins remnants interact with arterial wall endothelial cells, get retained in the subendothelial space, and elicit inflammatory responses, thereby accelerating atherogenesis.

Despite the clear association between high triglyceride levels and increased cardiovascular disease risk, intervention trials targeting triglyceride reduction have produced mixed results. We discuss a range of triglyceride-lowering agents, from fibrates to omega-3 fatty acids, with a focus on their mechanism of action, efficacy, and major clinical trial outcomes. Notably, the role of newer agents, such as angiopoietin-like protein 3 and apolipoprotein C3 inhibitors, is also explored.

We highlight the challenges and controversies, including the ongoing debate on the causal role of triglyceride in atherosclerosis and the discordant outcomes of recent clinical trials. The potential confounding effects of associated risk factors, such as elevated apolipoprotein B, insulin resistance, and metabolic syndrome, are considered.

In conclusion, this review underscores the importance of a nuanced approach to understanding the role of triglycerides in atherosclerosis and their potential as a therapeutic target. Further research is needed to unravel the complex interplay between triglycerides, triglyceride-rich lipoproteins, and associated factors in atherosclerosis pathogenesis and refine triglyceride-targeted therapeutic strategies.

## 1 INTRODUCTION

Triglycerides, the most abundant lipid class in nature, are an energy reservoir storing surplus calories ingested from our diet [1]. Structurally, they comprise a glycerol backbone esterified to three fatty acids, forming a molecule both simple and yet profoundly influential on human health. Elevated levels of serum triglycerides, or hypertriglyceridemia (>150 mg/dL), have long been associated with an array of conditions, including obesity, insulin resistance, and metabolic syndrome [2]. However, in the realm of cardiovascular health and atherosclerotic disease, the role of triglycerides has come under focused scrutiny [2. 3].

Atherosclerosis, a chronic and progressive inflammatory disease, is characterized by the accumulation of lipids and fibrous elements in the large arteries [4]. This devastating pathological process underpins the majority of cardiovascular diseases (CVDs) and remains the leading cause of mortality globally. Atherogenesis traditionally has been ascribed to the nefarious impact of elevated low-density lipoprotein cholesterol (LDL-C) [5]. Yet, a growing body of evidence suggests that triglycerides are not mere bystanders in this process but may play a significant, direct role in the initiation and progression of atherosclerosis [6].

The past two decades have seen remarkable advancements in lipid-lowering therapies, primarily targeted toward reducing LDL-C levels. Statins, the mainstay of lipid-lowering therapy, alongside newer agents, such as PCSK9 inhibitors, have reduced the burden of atherosclerotic CVD [7]. However, despite these interventions, a considerable residual cardiovascular risk remains. It is increasingly recognized that this residual risk may be attributed, at least in part, to elevated triglyceride levels, particularly in the era of rising obesity and metabolic syndrome prevalence [8-10].

Current pharmacotherapeutic options for hypertriglyceridemia include fibrates, niacin, and omega-3 fatty acids, each with its own benefits and limitations [11]. However, novel strategies aimed at targeting triglyceride-rich lipoproteins (TRLs) and their remnants are emerging, offering potential promise in addressing the unmet need in this domain. This review will delve into the intriguing world of triglycerides, exploring their metabolic pathways, their role in the pathogenesis of atherosclerosis, the current state of triglyceride-lowering therapy, and, importantly, the vanguard of emerging pharmacological strategies that may change our approach to managing triglyceride levels and, in turn, cardiovascular risk. In doing so, we hope to shed light on the intricate role of this pivotal lipid class and generate meaningful discussion on its evolving place in cardiovascular therapeutics.

## 2 TRIGLYCERIDE METABOLISM

Triglycerides represent a finely orchestrated symphony of energy storage and transport in the human body. Primarily synthesized in the liver, these energy-rich molecules are elegantly constructed around a simple glycerol backbone, to which three fatty acids are esterified [1]. This design, elegant in its simplicity, belies a complex metabolic pathway with profound implications for health and disease.

**Fig. (1):**
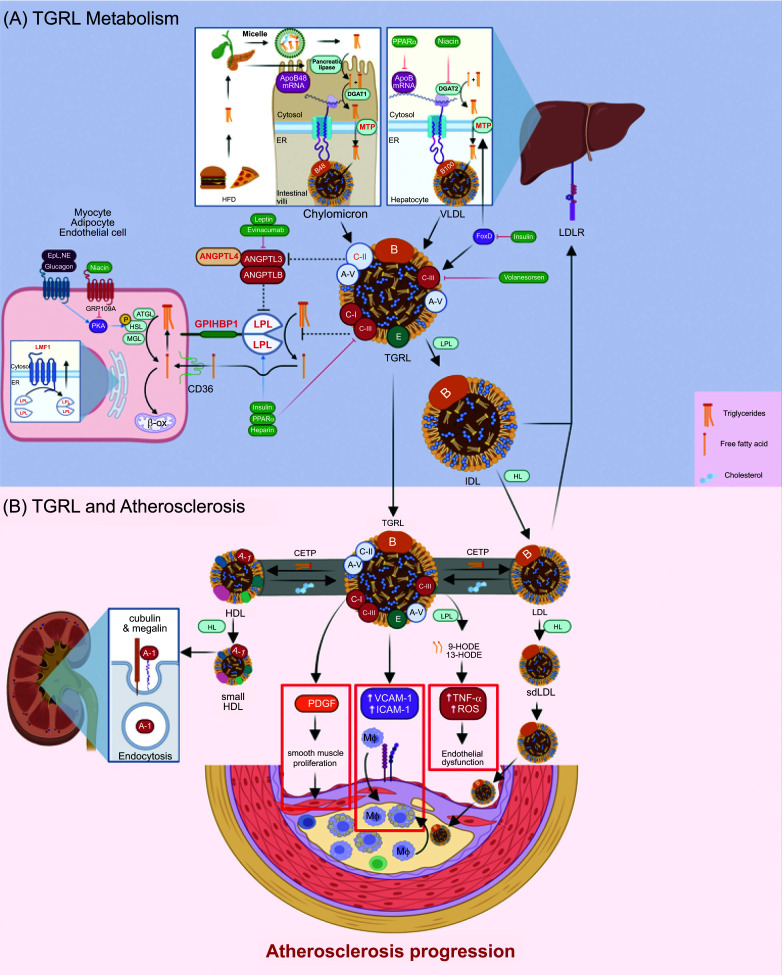
Triglycerides-rich lipoproteins (TGRL) carry the exogenously derived triglycerides in the form of chylomicrons in the fed state and the endogenously hepatically produced triglycerides in the form of very low-density lipoprotein **(A)**. While their main function is to carry the insoluble triglycerides as an energy source to peripheral tissues, their small size makes them readily accessible to subendothelial tissue, propagating atherosclerosis through upregulation of inflammation, endothelial dysfunction, and smooth muscle proliferation. To add insult to injury, TGRL exchanges triglycerides for cholesterol with two other lipoproteins, high-density lipoprotein and low-density lipoprotein, making triglycerides-rich HDL and LDL that further transform into a small, dense version of HDL and LDL. These small and dense lipoproteins further complicate the process of atherosclerosis, as sdLDL is far more atherogenic, while the kidneys degrade the leftover apoA-1 from the dense HDL **(B)**.

The metabolic journey of triglycerides begins in earnest with dietary absorption in the small intestine. Here, fat from the diet is emulsified by bile salts and hydrolyzed by pancreatic enzymes to form monoglycerides and free fatty acids [12-14]. These are re-esterified in the enterocytes to form triglycerides and packaged into chylomicrons, the largest and least dense lipoproteins (Fig. **1A**). Chylomicrons are then released into the lymphatic system and eventually enter the bloodstream *via* the thoracic duct, bypassing the portal vein [12]. Apolipoprotein B-48 (ApoB-48) tags each chylomicron particle and serves as their unique identifier [15].

Meanwhile, endogenous triglycerides are synthesized in the liver through a process involving the esterification of glycerol with fatty acids derived from dietary sources or de novo lipogenesis. These triglycerides are packaged into very low-density lipoprotein (VLDL) particles and released into circulation [16, 17]. Contrary to chylomicrons, apolipoprotein B-100 (ApoB-100) is the identifier found on the surface of each VLDL particle [15]. Both chylomicrons and VLDLs, the principal triglyceride-rich lipoproteins (TRLs), serve as vehicles to transport triglycerides to peripheral tissues, such as adipose tissue and skeletal muscle, for storage or energy use.

The hydrolysis of triglycerides within TRLs is mediated by the enzyme lipoprotein lipase (LPL), which is anchored to the vascular endothelium in various tissues, including adipocytes and muscle tissue [18]. LPL activity liberates free fatty acids, which are taken up by the surrounding tissues, either to be stored in the form of triglycerides in adipocytes or oxidized for energy production in muscle cells [18, 19]. LPL is tightly controlled, and adaptive alterations in activity are regulated by the interplay of insulin responses and the moderating effects of various hormones and proteins [20]. In a postprandial state, for example, higher insulin levels upregulate LPL gene expression and activity in adipocytes to facilitate the storage of dietary lipids. Different apolipoproteins on the surface of TRLs have distinct effects on LPL. Apolipoprotein C-II (apoC-II) and apoA-5 enhance LPL activity, while apolipoproteins C-I (apoC-I) and C-III (apoC-III) decrease it [21, 22].

Upon hydrolysis, TRLs are transformed into smaller, denser remnant particles that are enriched in cholesterol [23]. Clearance of these remnants from circulation is primarily mediated by the liver [24]. Recognition and uptake of these particles involve specific interactions with hepatic lipoprotein receptors, such as the low-density lipoprotein receptor (LDL-R) and the LDL receptor-related protein 1 (LRP1), which identify apolipoprotein on the surface of remnants, such as apoB-100 and ApoE [25-27].

When the body senses an energy deficit, such as during fasting or prolonged physical activity, it signals for the mobilization of this stored energy. The process begins with the hydrolysis of triglycerides, orchestrated by key enzymes, hormone-sensitive lipase (HSL), and adipose triglyceride lipase (ATGL) [28]. This enzymatic action breaks down the triglycerides into glycerol and free fatty acids, marking the first step in the liberation of stored energy. The journey of these liberated molecules is a testament to the body's ability to manage its resources. Glycerol, for instance, heads toward the liver, where it serves as a substrate for gluconeogenesis, contributing to the maintenance of blood glucose levels during fasting. The free fatty acids, on the other hand, embark on a journey through the bloodstream, reaching various tissues. Here, they undergo β-oxidation, generating acetyl-CoA, a crucial player in the energy-producing citric acid cycle in the mitochondria [29]. In times of prolonged fasting or carbohydrate shortage, the liver crafts these acetyl-CoA molecules into ketone bodies, providing an alternative energy source for the brain and other organs.

## 3 THE ROLE OF GENETICS IN TRIGLYCERIDE LEVELS:

Of the key players in triglyceride metabolism, LPL stands out. Mutations in the LPL gene can lead to LPL deficiency, a condition marked by severe hypertriglyceridemia due to impaired clearance of TRLs. One example of such mutation is in Glycosylphosphatidylinositol-anchored HDL-binding protein 1 (GPIHBP1) [30]. GPHBP1 functions as a transporter for LPL, escorting the enzyme from its place of production to the endothelial surface and facilitating its attachment to endothelial cells in capillaries [30]. In humans, homozygous GPIHBP1 variants present with familial chylomicron syndrome (FCS). Familial combined hyperlipidemia, another genetic disorder leading to elevated triglyceride levels, is thought to result from the overproduction of VLDL [31].

Beyond LPL, mutations in genes that modulate LPL activity can have effects on triglyceride levels. For instance, mutations in the APOC2 gene can impair LPL activity, leading to elevated triglyceride levels [32]. Similarly, mutations in the APOA5 gene can result in hypertriglyceridemia [22]. Recent studies propose that apoA-V reduces triglyceride levels by counteracting LPL inhibition induced by angiopoietin-related protein 3 (ANGPTL3) and ANGPTL8 [22].

On the other hand, certain genetic variations can confer a protective effect. ANGPTL4 is expressed in several tissues, including muscle, adipose tissue, and liver, and inhibits LPL activity in white adipose tissue in humans. Loss of function mutations in the ANGPTL4 gene have been associated with lower triglyceride levels and a reduced risk of coronary artery disease [33].

It is important to note that the genetic architecture of triglyceride levels is complex and polygenic, involving multiple genes, each contributing a small effect. Genome-wide association studies (GWAS) have identified several genetic loci associated with triglyceride levels, underscoring the polygenic nature of this trait.

## 4 EVIDENCE LINKING TRIGLYCERIDES TO ATHEROSCLEROSIS

Numerous epidemiological studies have demonstrated a positive correlation between elevated plasma triglyceride levels and increased cardiovascular risk. For example, in the seminal Framingham Heart Study, elevated triglyceride levels were independently associated with an increased incidence of coronary heart disease (CHD) in both men and women over a 12-year follow-up period [33]. In the Copenhagen City Heart Study, non-fasting triglyceride levels were found to be a significant predictor of myocardial infarction, ischemic heart disease, and overall mortality, even after adjusting for other known cardiovascular risk factors [34]. In a meta-analysis conducted by Sarwar *et al*. (2007) involving 262,525 participants from 29 Western prospective studies, individuals with higher triglyceride levels had a 72% increased risk of CHD events compared to those with lower levels, after adjusting for HDL-C and other risk factors [35].

### 4.1 Triglyceride and Atherosclerosis: Mechanistic Insight

Atherosclerosis is a chronic inflammatory disease characterized by the buildup of lipids, immune cells, and fibrous tissue within the arterial wall, leading to the formation of atherosclerotic plaques [36]. This process typically begins with endothelial injury or dysfunction, which facilitates the infiltration of LDL particles into the arterial intima. Once within the intima, LDL particles can become oxidized, triggering an inflammatory response that recruits immune cells to the site of injury [2, 36, 37]. These immune cells, particularly macrophages, engulf the oxidized LDL particles, transforming into foam cells and initiating the formation of the fatty streak, the earliest visible lesion in atherosclerosis (Fig. **1B**).

For many years, the role of triglycerides in atherosclerosis was overshadowed by the focus on cholesterol, specifically LDL-C and high-density lipoprotein cholesterol (HDL-C). However, emerging evidence indicates that triglycerides and the lipoproteins that carry them may play a significant role in the pathogenesis of atherosclerosis [34]. TRLs and their remnants are particularly atherogenic for several reasons. Firstly, they are small and dense enough to permeate the arterial endothelium, and their high lipid content makes them prone to oxidative modification and retention within the arterial wall [34]. Secondly, the apolipoproteins on the surface of these particles, such as ApoC-III and ApoB-100, can promote inflammation, endothelial dysfunction, and thrombosis, key steps in atherogenesis. It has been shown that TRLs can activate endothelial cells to express adhesion molecules, such as VCAM-1 and ICAM-1, promoting the recruitment and adhesion of monocytes to the endothelium [38]. TRLs can also contribute to the formation of foam cells, a hallmark of atherosclerotic plaques. TRL remnants can penetrate the subendothelial space, where they can be taken up by macrophages *via* scavenger receptors, leading to the accumulation of cholesterol esters and the formation of foam cells [39]. TRLs have been reported to stimulate the proliferation of smooth muscle cells, which contribute to plaque progression. For example, Doi *et al.* (2000) demonstrated that chylomicron remnants induce the proliferation of smooth muscle cells in the arterial wall, possibly *via* the activation of platelet-derived growth factor (PDGF) signaling [40].

Beyond their role in fostering an increased cardiovascular risk through the production of TRLs, elevated triglycerides also augment the production of LDL particles, thus amplifying their atherogenic potential. Indeed, the interplay between TRL and LDL particles is a key aspect of lipid metabolism that can significantly influence atherogenic risk. There is an increased exchange of triglycerides and cholesteryl esters between TRLs and LDL particles, mediated by the enzyme cholesteryl ester transfer protein (CETP) [41, 42]. This exchange process results in triglyceride-enriched LDL particles. These triglyceride-enriched LDL particles are then substrates for hepatic lipase, an enzyme that hydrolyzes the triglycerides, leading to the formation of smaller, denser LDL particles, often termed small dense LDL (sdLDL) [43]. Importantly, sdLDL particles are more atherogenic than their larger, buoyant counterparts. They are more susceptible to oxidative modification, have a reduced affinity for the LDL receptor, and thus, have a longer circulation time, all of which increase their likelihood of contributing to the formation of atherosclerotic plaques. Moreover, due to their small size, sdLDL particles can more easily penetrate the endothelium and get trapped in the arterial wall [44]. In this way, elevated triglyceride levels, through the increased production of TRLs and the subsequent alterations in LDL metabolism, can lead to an increased LDL particle number, specifically the more atherogenic sdLDL particles, thereby contributing to the development and progression of atherosclerosis.

### 4.2 Current Pharmacotherapies Targeting Triglyceride Metabolism

Triglyceride-lowering therapies have evolved substantially over the years. Various classes of drugs, including fibrates, niacin, omega-3 fatty acids, and statins, have been employed to modulate triglyceride levels and mitigate the associated cardiovascular risk. In this section, we delve into the mechanisms of these current therapies and examine key clinical trials that highlight their therapeutic effects and outcomes (Tables **1** and **2**).

### 4.3 Fibrates

Fibrates are synthetic agonists of peroxisome proliferator-activated receptor alpha (PPARα), which are predominantly found in the liver, muscle, and kidney. After activation, PPARα forms a heterodimer with retinoid X receptor (RXR) and translocates to the nucleus. Here, it binds to the promoter region of several genes containing the AGGTCA-motif response element known as PPARα Response Element (PPRE). This binding leads to the activation or inhibition of critical target genes involved in fatty acid metabolism, ultimately reducing triglyceride levels through enhanced clearance and reduced synthesis. It also increases the clearance of TRLs by upregulating LPL and downregulating ApoC-III. Moreover, PPARα reduces the expression of ApoB and VLDL, contributing to decreased triglyceride production [45]. In addition, the PPARα-RXR complex enhances HDL levels by upregulating ApoA-I and ApoA-II. This effect on HDL, combined with the upregulation of several other genes, such as ATP-binding cassette transporter A1 (ABCA1) and scavenger receptor class B type I (SR-BI), contributes to the process of reverse cholesterol transport. This process shuttles cholesterol from macrophages back to hepatocytes, potentially mitigating the development of atherosclerotic plaques.

Several key clinical trials have evaluated the efficacy of fibrates in managing lipid levels and cardiovascular risk:

- **FIELD** (Fenofibrate Intervention and Event Lowering in Diabetes) study: This study randomized 9,795 patients with type 2 diabetes to fenofibrate or placebo. Over a mean follow-up of 5 years, fenofibrate did not significantly reduce the risk of the primary outcome of coronary events (HR 0.89; 95% CI, 0.75-1.05; *p=*0.16). However, it did reduce total cardiovascular events, mainly due to fewer non-fatal myocardial infarctions (4.5% *vs.* 5.9%; *p=*0.01) and revascularizations (5.2% *vs.* 6.6%; *p=*0.002) [46].
- **VA-HIT** (Veterans Affairs High-Density Lipoprotein Intervention Trial): This trial involved 2,531 men with low HDL-C levels and randomized them to gemfibrozil or placebo. After 5.1 years, gemfibrozil reduced the incidence of the primary endpoint (nonfatal myocardial infarction or coronary death) by 22% (17.3% *vs.* 22.7%; RR 0.78; 95% CI, 0.65-0.93; *p=*0.006) [47].
- **HHS** (Helsinki Heart Study): In this trial, 4,081 asymptomatic men with dyslipidemia were randomized to gemfibrozil or placebo. After 5 years, gemfibrozil led to a 34% reduction in the incidence of coronary heart disease (2.7% *vs.* 4.1%; RR 0.66; 95% CI, 0.44-0.98; *p=*0.04) [48].
- **ACCORD Lipid trial:** This study randomized 5,518 patients with type 2 diabetes to fenofibrate plus simvastatin or simvastatin alone. After a mean follow-up of 4.7 years, the combination of fenofibrate and simvastatin did not significantly reduce the rate of fatal cardiovascular events, nonfatal myocardial infarction, or nonfatal stroke compared to simvastatin alone (2.2% *vs.* 2.4%; HR 0.92; 95% CI, 0.79-1.08; *p=*0.32) [49].

### 4.4 Niacin

Niacin, or vitamin B3, has a long history of use in the management of dyslipidemia. Its primary mechanism of action in lipid-lowering involves inhibiting diacylglycerol acyltransferase 2 (DGAT2), an enzyme involved in triglyceride synthesis, as well as reducing the hepatic uptake of free fatty acids [50]. Niacin also has the unique ability among lipid-lowering therapies to significantly raise HDL-C levels and lower LDL-C levels. Several clinical trials have been conducted to evaluate the impact of niacin on lipid levels and cardiovascular outcomes:

- **Coronary Drug Project (CDP):** This trial randomized 8,341 men with a history of myocardial infarction to niacin or placebo. After 6 years of follow-up, niacin led to a significant reduction in nonfatal myocardial infarction (7.8% *vs.* 12.2%, *p<*0.005). Furthermore, 15-year follow-up data showed a modest reduction in total mortality (52% *vs.* 58%, *p=*0.0004) [51].
- **AIM-HIGH (Atherothrombosis Intervention in Metabolic Syndrome with Low HDL/High Triglycerides and Impact on Global Health Outcomes) trial:** This trial included 3,414 patients with cardiovascular disease and dyslipidemia despite LDL-C control on statin therapy. Patients were randomized to extended-release niacin or placebo, both on top of simvastatin therapy. The trial was stopped early after a median follow-up of 3 years due to lack of efficacy. There was no significant difference in the primary endpoint, a composite of cardiovascular death, nonfatal myocardial infarction, ischemic stroke, hospitalization for the acute coronary syndrome, or symptom-driven coronary or cerebrovascular revascularization (16.4% *vs.* 16.2%; HR 1.02; 95% CI, 0.87-1.21; *p=*0.79) [52].
- **HPS2-THRIVE (Heart Protection Study 2–Treatment of HDL to Reduce the Incidence of Vascular Events) trial:** This large trial randomized 25,673 patients with atherosclerotic vascular disease to extended-release niacin/laropiprant or placebo, both on top of statin therapy. After a median follow-up of 3.9 years, there was no significant difference in the primary endpoint, a composite of coronary deaths, non-fatal myocardial infarctions, strokes, or revascularizations (13.2% *vs.* 13.7%; RR 0.96; 95% CI, 0.90-1.03; *p=*0.29) [53].

These trials have provided important insights into the effects of niacin on cardiovascular outcomes. Despite its effectiveness in modifying lipid profiles, niacin has not consistently demonstrated a beneficial effect on cardiovascular events when added to statin therapy, leading to a re-evaluation of its role in the management of dyslipidemia.

### 4.5 Omega-3 Fatty Acids

Omega-3 fatty acids, specifically eicosapentaenoic acid (EPA) and docosahexaenoic acid (DHA), have demonstrated efficacy in lowering triglyceride levels. They exert their effects by reducing hepatic production and secretion of VLDL particles [54]. Several pivotal clinical trials have assessed the effects of omega-3 fatty acids on lipid levels and cardiovascular outcomes:

- **JELIS (Japan EPA Lipid Intervention Study) trial:** This was a large, open-label study that randomized 18,645 patients with hypercholesterolemia to statin therapy with or without EPA. After a median follow-up of 4.6 years, the addition of EPA led to a 19% reduction in major coronary events (2.8% *vs.* 3.5%; HR 0.81; 95% CI, 0.69-0.95; *p=*0.011) [55].
- **REDUCE-IT (Reduction of Cardiovascular Events with Icosapent Ethyl–Intervention Trial):** This trial randomized 8,179 patients with elevated triglyceride levels despite statin therapy to high-dose EPA (icosapent ethyl) or placebo. After a median follow-up of 4.9 years, icosapent ethyl significantly reduced the primary endpoint, a composite of cardiovascular death, nonfatal myocardial infarction, nonfatal stroke, coronary revascularization, or unstable angina, by 25% (17.2% *vs.* 22.0%; HR 0.75; 95% CI, 0.68-0.83; *p<*0.001) [11].
- **STRENGTH (STatin Residual Risk Reduction With EpaNova in HiGh CV Risk PatienTs With Hypertriglyceridemia):** This trial randomized 13,078 patients with high cardiovascular risk and elevated triglyceride levels despite statin therapy to a carboxylic acid formulation of EPA/DHA or corn oil placebo. The trial was halted early for futility after a median follow-up of 42 months. There was no significant difference in the primary composite endpoint of cardiovascular death, nonfatal myocardial infarction, nonfatal stroke, coronary revascularization, or unstable angina (12.0% *vs.* 12.2%; HR 0.99; 95% CI, 0.90-1.09; *p=*0.84) [56].

These trials highlight the potential cardiovascular benefits of omega-3 fatty acids, particularly high-dose EPA, in patients with elevated triglyceride levels. However, the findings also underscore the need for further investigation to clarify the role of DHA and the optimal formulation of omega-3 fatty acids for cardiovascular risk reduction.

The management of hypertriglyceridemia has been refined over the years with the utilization of fibrates, niacin, and omega-3 fatty acids. Each of these classes of drugs, through their unique mechanisms of action, contributes to the modulation of lipid profiles and the mitigation of cardiovascular risk. However, as the trials reviewed above suggest, the efficacy of these agents in altering hard cardiovascular outcomes varies, with some therapies showing promise and others necessitating further study. This underlines the complexity of lipid metabolism and its interplay with atherosclerosis and cardiovascular disease. As we transition into the next section of this review, we will explore emerging pharmacotherapies that are currently under investigation, with a focus on their potential to our understanding of triglyceride metabolism and offer new avenues for the prevention and treatment of cardiovascular diseases.

### 4.6 Emerging Pharmacotherapies Targeting Triglyceride Metabolism

As we delve into the world of emerging pharmacotherapies targeting triglyceride metabolism, we encounter new mechanisms of action and novel therapeutic targets. These innovative strategies offer the promise of better controlling hypertriglyceridemia and, in turn, reducing cardiovascular risk.

### 4.7 Volanesorsen

Volanesorsen is an antisense oligonucleotide drug designed to target and degrade ApoC-III mRNA in the liver, which results in a decrease in the production of ApoC-III protein. This drug holds significant potential in modulating the role of ApoC-III in triglyceride metabolism and cardiovascular diseases.

ApoC-III plays a pivotal role in triglyceride metabolism. It is synthesized mainly in the liver and, to a lesser degree, in the intestines. Once synthesized, it becomes a component of chylomicrons and VLDL [57-59].

Volanesorsen, by reducing the production of ApoC-III, interferes with several mechanisms:

- **Enhancement of LPL Activity**: ApoC-III inhibits the activity of LPL. By lowering ApoC-III levels, volanesorsen relieves this inhibition, promoting LPL activity and the subsequent hydrolysis of triglycerides. This leads to decreased circulating triglyceride levels [22, 57-59].
- **Increased Hepatic Uptake of TRLs:** ApoC-III interferes with the hepatic uptake of triglyceride-rich remnants by disrupting their interaction with hepatic receptors, including LDL-R and LRP. This contributes to increased circulating triglyceride levels. By reducing ApoC-III levels, volanesorsen can facilitate the interaction of these remnants with their hepatic receptors, enhancing their clearance from circulation [22].
- **Potential Reduction in VLDL Secretion**: While still under investigation, some studies suggest that ApoC-III may promote the hepatic secretion of VLDL, contributing to hypertriglyceridemia. By lowering ApoC-III production, volanesorsen might potentially reduce VLDL secretion.
- **Potential Impact on Atherosclerosis:** ApoC-III has been implicated in promoting inflammation and endothelial dysfunction, both critical processes in atherogenesis. Elevated levels of ApoC-III in the circulation have been associated with increased cardiovascular risk. By reducing ApoC-III levels, volanesorsen may help mitigate these atherosclerotic processes [22, 57-59].

Clinical trials have demonstrated that volanesorsen can significantly reduce circulating ApoC-III and triglyceride levels. The APPROACH trial showed a mean reduction in triglyceride levels of approximately 77% in patients with familial chylomicronemia syndrome [60]. In the COMPASS trial, patients with severe hypertriglyceridemia showed a mean reduction of about 71% [61]. These findings suggest that volanesorsen could be a promising therapeutic option for patients with severe hypertriglyceridemia and potentially those at high risk for atherosclerotic cardiovascular disease.

### 4.8 Pemafibrate

Pemafibrate is a novel selective PPAR-α modulator (SPPARMα). In comparison to traditional fibrates like fenofibrate and gemfibrozil, Pemafibrate has a much higher selectivity and potency for PPAR-α, which results in more pronounced effects on triglyceride metabolism with potentially fewer side effects [59, 62, 63].

Upon activation by Pemafibrate, PPAR-α modulates lipid metabolism through several mechanisms [59, 62, 63]:

- **Enhancement of LPL Activity:** PPAR-α activation leads to increased expression of LPL, an enzyme responsible for hydrolyzing triglycerides in chylomicrons and VLDL. This results in a reduction of circulating triglyceride levels.
- **Increased Fatty Acid Oxidation:** PPAR-α activation upregulates the expression of genes involved in fatty acid transport and oxidation in the liver and skeletal muscle, which leads to a decrease in the availability of free fatty acids for triglyceride synthesis.
- **Reduction of VLDL Production:** PPAR-α activation reduces the hepatic production and secretion of VLDL, a major carrier of triglycerides in the circulation.
- **Potential Effects on HDL Metabolism and Inflammation:** Pemafibrate, through PPAR-α activation, increases the expression of ApoA-I and ApoA-II, major components of HDL. This could potentially lead to increased HDL levels and enhanced reverse cholesterol transport, a process by which excess cholesterol is transported from peripheral tissues back to the liver for excretion. Furthermore, PPAR-α activation has been associated with anti-inflammatory effects, which may confer additional benefits in the context of atherosclerosis.

Clinical trials have demonstrated the efficacy of Pemafibrate in significantly reducing triglyceride levels and increasing HDL-C levels. The PROMINENT study, however, reported that Pemafibrate failed to lower cardiovascular mortality despite its effective triglyceride, VLDL-C, and remnant cholesterol-lowering properties [64].

### 4.9 Evinacumab

Evinacumab is a fully human monoclonal antibody that inhibits ANGPTL3. ANGPTL3, predominantly expressed and secreted by the liver, inhibits LPL and endothelial lipase (EL). Therefore, the inhibition of ANGPTL3 by evinacumab can lead to increased activity of LPL and EL and, thus, a reduction in circulating levels of triglycerides and LDL-C [59, 65, 66].

By inhibiting ANGPTL3, Evinacumab interferes with several mechanisms [59, 65, 66]:

- **Enhancement of LPL Activity:** The inhibition of ANGPTL3 by evinacumab leads to increased LPL activity. Increased LPL activity results in lower circulating levels of triglycerides.
- **Increased EL Activity:** ANGPTL3 also inhibits EL, an enzyme that hydrolyzes phospholipids in HDL, leading to the production of smaller, denser HDL particles. The inhibition of ANGPTL3 by evinacumab leads to increased EL activity, potentially affecting HDL metabolism. However, the clinical implications of this effect are not entirely clear and require further investigation.
- **Reduction of VLDL and LDL Production:** ANGPTL3 has been implicated in the hepatic production of VLDL and LDL. By inhibiting ANGPTL3, evinacumab may reduce the hepatic output of these atherogenic lipoproteins, contributing to lower circulating levels of LDL-C.

The effectiveness of evinacumab in lowering LDL-C levels has been demonstrated in patients with homozygous familial hypercholesterolemia, a group of individuals who often have extreme elevations in LDL-C levels that are resistant to standard lipid-lowering therapies. In the ELIPSE HoFH trial, evinacumab led to a significant reduction in LDL-C levels beyond what was achieved with other lipid-lowering therapies [67]. Ongoing trials are investigating the effects of evinacumab in broader patient populations and its impact on cardiovascular outcomes.

## CONCLUSION

In conclusion, our understanding and management of hypertriglyceridemia and related cardiovascular risk have come a long way. Traditional pharmacotherapies, including fibrates, niacin, and omega-3 fatty acids, have provided significant insights into the complex biology of lipid metabolism. However, as reflected in the evidence from clinical trials, they have shown variable results in terms of cardiovascular outcomes, underscoring the need for further advancement in this area.

The exploration of new targets, such as ApoC-III, PPAR-α, and ANGPTL3, is an exciting development in this field. The development of novel therapies targeting these pathways, including Volanesorsen, Pemafibrate, and Evinacumab, respectively, offers the potential to further optimize lipid management. Through their unique mechanisms of action, these agents have shown promising results in early-phase trials, demonstrating both efficacy in lowering triglyceride levels and potential benefits in terms of cardiovascular outcomes.

However, it is important to remember that the full impact of these emerging therapies on cardiovascular risk reduction is yet to be established. Furthermore, the implications of long-term use of these agents, including potential side effects and the impact on other aspects of metabolic health, also require thorough investigation.

As we progress in our understanding of lipid biology and its interplay with cardiovascular disease, the development and evaluation of these novel therapies will play a crucial role. The journey toward optimal management of hypertriglyceridemia is ongoing, and these emerging therapies signify promising strides in this direction. This is an exciting time in the field of lipidology as we continue to explore and refine our therapeutic options for the management of dyslipidemia and the prevention of cardiovascular disease. The coming years will undoubtedly bring further advances and challenges in this important area of cardiovascular medicine.

## Figures and Tables

**Table 1:** Genetic mutations affecting lipid metabolism and cardiovascular risk.

**Gene**	**Function of Normal Gene**	**Impact of Mutation**
LPL (Lipoprotein Lipase)	Hydrolyzes triglycerides in TRLs, enabling the release and uptake of free fatty acids by tissues	Mutations can lead to LPL deficiency, resulting in severe hypertriglyceridemia due to impaired clearance of TRLs
APOC2 (Apolipoprotein C-II)	Serves as a cofactor for LPL	Mutations can impair LPL activity, leading to elevated triglyceride levels
APOA5 (Apolipoprotein A-V)	Enhances LPL-mediated triglyceride hydrolysis	Mutations can result in hypertriglyceridemia
ANGPTL4 (Angiopoietin-Like 4)	Inhibits LPL activity	Loss-of-function mutations have been associated with lower triglyceride levels and a reduced risk of coronary artery disease
LMF1 (Lipase Maturation Factor 1)	Involved in the maturation of LPL	Mutations can lead to combined lipase deficiency, resulting in elevated triglyceride levels
GPIHBP1 (Glycosylphosphatidylinositol Anchored High-Density Lipoprotein Binding Protein 1)	Binds to LPL and anchors it to the capillary endothelium	Mutations can lead to LPL mislocalization and hypertriglyceridemia

**Table 2:** Lipoproteins, associated proteins, and receptors in lipid metabolism.

**Lipoprotein**	**Associated Proteins**	**Receptor**
Chylomicron	Apolipoprotein B-48 (ApoB-48), Apolipoprotein C-II (ApoC-II), Apolipoprotein E (ApoE)	Low-density lipoprotein receptor (LDLR), LDL receptor-related protein (LRP)
Very Low-Density Lipoprotein (VLDL)	Apolipoprotein B-100 (ApoB-100), Apolipoprotein C-II (ApoC-II), Apolipoprotein E (ApoE)	Low-density lipoprotein receptor (LDLR)
Intermediate-Density Lipoprotein (IDL)	Apolipoprotein B-100 (ApoB-100), Apolipoprotein E (ApoE)	Low-density lipoprotein receptor (LDLR), LDL receptor-related protein (LRP)
Low-Density Lipoprotein (LDL)	Apolipoprotein B-100 (ApoB-100)	Low-density lipoprotein receptor (LDLR)
High-Density Lipoprotein (HDL)	Apolipoprotein A-I (ApoA-I), Apolipoprotein A-II (ApoA-II)	Scavenger Receptor Class B type 1 (SR-B1)

## LIST OF ABBREVIATIONS

LDL-C: Low-density lipoprotein cholesterol
CVDs: Cardiovascular diseases
TRLs: Triglyceride-rich lipoproteins
VLDL: Very low-density lipoprotein
LPL: Lipoprotein lipase
ApoB-48: Apolipoprotein B-48
ApoB-100: Apolipoprotein B-100
apoC-II: Apolipoprotein C-II
apoA-5: Apolipoprotein A-5
apoC-I: Apolipoprotein C-I
apoC-III: Apolipoprotein C-III
ANGPTL3: Angiopoietin-related protein 3
ANGPTL8: Angiopoietin-related protein 8
LDL-R: Low-density lipoprotein receptor
LRP1: Low-density lipoprotein receptor-related protein 1
HSL: Hormone-sensitive lipase
ATGL: Adipose triglyceride lipase
HDL-C: High-density lipoprotein cholesterol
GWAS: Genome-wide association studies
CHD: Coronary heart disease
VCAM-1: Vascular cell adhesion molecule 1
ICAM-1: Intercellular adhesion molecule 1
PDGF: Platelet-derived growth factor
CETP: Cholesteryl ester transfer protein
sdLDL: Small dense LDL
EPA: Eicosapentaenoic acid
DHA: Docosahexaenoic acid
DGAT2: Diacylglycerol acyltransferase 2
PPARα: Peroxisome proliferator-activated receptor alpha
RXR: Retinoid X receptor
ABCA1: ATP-binding cassette transporter A1
SR-BI: Scavenger receptor class B type I
GPIHBP1: Glycosylphosphatidylinositol-anchored HDL-binding protein 1
FCS: Familial chylomicronemia syndrome
SPPARMα: Selective PPAR-α modulator
EL: Endothelial lipase
